# Towards fluid human-agent collaboration: From dynamic collaboration patterns to models of theory of mind reasoning

**DOI:** 10.3389/frobt.2025.1532693

**Published:** 2025-08-01

**Authors:** Florian Schröder, Fabian Heinrich, Stefan Kopp

**Affiliations:** Social Cognitive Systems Group, Faculty of Technology/CITEC, Bielefeld University, Bielefeld, Germany

**Keywords:** fluid collaboration, theory of mind, dynamic mentalizing, human-agent collaboration, action-oriented reasoning, cooperative cuisine, real-time adaptation, collaborative agents

## Abstract

Collaborating in real-life situations rarely follows predefined roles or plans, but is established on the fly and flexibly coordinated by the interacting agents. We introduce the notion of fluid collaboration (FC), marked by frequent changes of the tasks partners assume or the resources they consume in response to varying requirements or affordances of the environment, tasks, or other agents. FC thus necessitates dynamic, action-oriented Theory of Mind reasoning to enable agents to continuously infer and adapt to others’ intentions and beliefs in real-time. In this paper, we discuss how FC can be enabled in human-agent collaboration. We introduce Cooperative Cuisine, an interactive environment inspired by the game *Overcooked!* that facilitates human-human and human-agent collaboration in dynamic settings. We report results of an empirical study on human-human collaboration in CoCu, showing how FC can be measured empirically and that humans naturally engage in dynamically established collaboration patterns with minimal explicit communication and relying on efficient mentalizing. We then present an approach to develop artificial agents that can effectively participate in FC. Specifically, we argue for a model of dynamic mentalizing under computational constraints and integrated with action planning. We present first steps in this direction by addressing resource-rational and action-driven ToM reasoning.

## 1 Introduction

Human collaboration in everyday, real-life scenarios is characterized by flexible behavior and adaptive coordination. For example, consider working together with a friend in the kitchen to prepare a meal. While both of you share a common goal, the subtasks carried out by each agent will vary considerably and may involve different degrees of interdependence (e.g., separately preparing ingredients vs. kneading and flouring a dough together). Who is in charge of what rarely will be determined at the outset and, if so, only partially or for a limited period of time. Rather, there will be rapid changes to these tasks and the overall cooperative activity, in response to arising task needs (e.g., needing more flour), events in the environment (e.g., a pot boiling over), or individual needs of other agents (e.g., the partner temporarily needing a helping hand). These adaptations can be reactive as well as proactive, and they can be signaled implicitly via behavioral changes (e.g., suddenly turning to the oven) or can be explicitly coordinated by means of situated communication. We refer to this highly adaptive way of working together as *fluid collaboration* (FC, henceforth) – a mode of interaction that is natural and intuitive for humans but in stark contrast to many forms of teamwork in professional environments, where team structures, individual roles, assignments, or interaction protocols are largely predetermined and optimized for the specific team task.

In the literature, the term fluid collaboration has been used to denote modes of collaboration that are characterized by frequent, fast transitions. This dates back to Pelletier (1970), who described how children in Indigenous communities, in contrast to Western culture, learned through fluid integration into daily community life, without formal organization or structure. This approach aligns with the concept of Learning by Observing and Pitching In (Rogoff, 2014). Ruvalcaba and Rogoff (2022) studied the collaboration of children through the concept of fluid synchrony, corresponding to enhanced abilities for anticipating each other’s contributions without proposals and leading to significantly better collaboration overall. Lissermann (2014) introduced the concept of fluid collaboration as the transition between different collaboration styles (individual work, tight and loose group work). Yamashita et al. (2011) used the term fluid (in contrast to fixed) collaboration to describe the flexible movement of players around a game tabletop. Others have used the term more widely for the smooth interplay of physical and virtual workspaces^1^ or breaking barriers between managerial and other staff^2^. The common underlying theme of all these notions is a shift from constant or rigid modes of collaboration to adaptive, dynamic collaboration patterns that emerge in real-world interaction.

**FIGURE 1:**
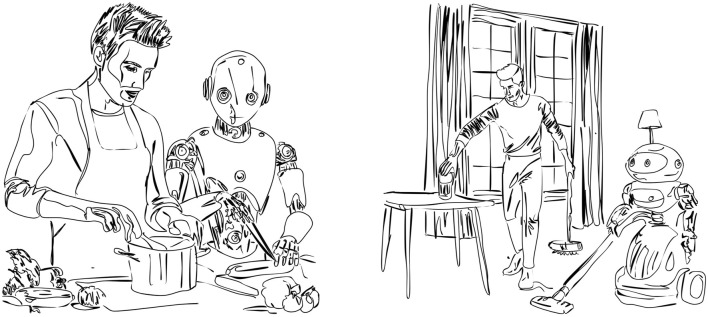
Illustration of real-world settings in which human-agent collaboration is fluid in that tasks and resources are assigned, shared, and re-adjusted in a highly dynamic, yet coordinated manner.

Despite its prominence in everyday scenarios like the aforementioned kitchen scenario, FC is beyond the capabilities of current AI-based collaborative agents or robots. Enabling it would in fact constitute a leap for human-agent interaction in settings in which humans (especially lay users) and AI-based agents differ considerably in their skills and abilities, but explicit negotiation and predetermination of roles or tasks is hardly feasible (as illustrated in Figure 1). In these situations, humans do not devise a detailed plan at the outset either, but identify and coordinate tasks as they arise in response to dynamic changes of the environment or varying uncertainty about each other’s behavior. This is possible due to our ability to swiftly employ a variety of coordination mechanisms (Espinosa et al., 2004; Malone and Crowston, 1994). Sometimes we point out our needs and negotiate our plans via natural language; sometimes we predict when another one requires assistance from observing their actions and inferring what they want to do next, what they ponder about, or what tools they are looking for to complete their task.

By and large, collaboration involves the coordination of resources and tasks, each of which may be distinctly assumed by (or assigned to) one or more agents for a certain period of time. Such assignments can constitute certain collaboration patterns. For example, in the cooking example, a straightforward resource-based pattern would be to decompose tasks into subtasks based on the equipment they utilize. Alternatively, an equally valid pattern would be to organize tasks around the parts of the meal that need to be prepared. Both patterns induce distinct assignments of emergent tasks and resources, thereby facilitating collaboration. In FC, however, those assignments along with the corresponding collaboration pattern become flexible and undergo frequent changes that need to be initiated, recognized, and coordinated among partners.

A fundamental capability underlying collaboration is Theory of Mind (ToM), also referred to as mentalizing–the ability to infer the mental states of others, such as intentions, goals, desires, or emotions (Premack and Woodruff, 1978; Baron-Cohen et al., 1985; Wimmer and Perner, 1983). Traditionally, ToM has been studied and modeled computationally as a form of offline inference from passive observation (e.g., in the classical Sally-Anne false belief test (Baron-Cohen et al., 1985)). However, FC requires active engagement and online participation based on continuous inferences of partners’ intentions or beliefs about the currently established patterns of collaboration or necessary changes to them. In consequence, FC requires abilities for online ToM reasoning that proceeds rapidly, in service of, and concurrent with task-oriented collaborative action. These forms of ToM are as of now not fully understood in humans, let alone successfully modeled computationally. As a result, seemingly simple collaborative interactions like jointly preparing a meal, which seem trivial and even enjoyable for humans, pose great challenges for the development of AI-based collaboration partners. Addressing them necessitates a comprehensive approach that seamlessly integrates perception, ToM, planning, communication, and acting in a unified framework for fluid collaboration agents.

In this paper, we present work towards this goal. We start by discussing related work in the next section. Then we confine the concept of fluid collaboration (FC) and propose a compositional definition that allows for characterizing and measuring crucial features of this kind of collaboration (e.g., intertwinement and fluidity). In Section 4, we propose a scenario for eliciting and studying FC between multiple humans and/or AI-based agents. We base this scenario on the collaborative computer game *Overcooked!* in which players have to prepare meals in a simulated kitchen environment with limited resources. We describe how we have extended this scenario to evoke and study (i.e., measure and model) FC in human-human interactions. Based on these data, we sharpen the concept of FC, analyze its emergence, and discuss takeaways for the design of FC-capable artificial agents. Finally, Section 5 presents foundational steps in developing such agents, with a focus on how to model the required forms of online ToM and how to combine ToM and planning to allow for fast and action-directed mentalizing.

## 2 Related work

### 2.1 Human collaboration and teamwork

Human collaboration has been studied in various forms, domains, or scales, including in organizations and work environments (Fisher and Dourish, 2004), research (Bukvova, 2010), education (Vangrieken et al., 2015), inter-organizational collaboration (Knoben and Oerlemans, 2006), and collaborations between industries (Ankrah and AL-Tabbaa, 2015). These fields, each vast on its own, do not deal with the kind of situated, task-oriented collaborations we consider here. In addition, these works do not consider fluid collaboration as they are mostly built on rather rigid structures and protocols. More fitting to the present work is the vast literature on teams, in which humans work closely and interdependently towards a shared goal (Salas et al., 2018; He et al., 2023; Mathieu et al., 2017). A distinguishing feature of effective teams is their ability to effortlessly distribute tasks between team members (Salas et al., 2005). Three core competencies have been identified–coordination, communication, and adaptability–as critical drivers of team success (Salas et al., 2018), with cohesive teams generally displaying better performance (Casey-Campbell and Martens, 2009; Grossman et al., 2022).

Team cognition refers to the collective knowledge structures and cognitive processes that emerge from and support coordinated team performance (Cooke et al., 2013). It encompasses how teams acquire, represent, share, and integrate information, enabling members to anticipate each other’s actions and respond adaptively during complex tasks, through mechanisms such as shared mental models, mentalizing, and communication. These mechanisms are deeply interconnected processes that enable effective collaboration in teams, leading to better team performance (Dechurch and Mesmer-Magnus, 2010). Shared mental models refer to the common understanding among team members about tasks, roles, and strategies, allowing for smooth coordination even with minimal explicit communication (Mathieu et al., 2000; Cooke et al., 2000; Mohammed et al., 2010).

A key concept proposed to analyze teamwork is the interdependence between the tasks performed by team members (Johnson and Bradshaw, 2021). Saavedra et al. (1993) analyzed group interactions through the theory of complex interdependence, emphasizing the interplay of task structure, feedback mechanisms, and goal alignment. Johnson et al. (2014) differentiate between hard and soft dependencies, where the former refers to structural constraints (e.g., role dependencies) that have to be managed as a team, while the latter are more opportunistic and refer to flexible opportunities for coordination that can be exploited by the team (Johnson et al., 2020). Task interdependence has a strong impact on effective communication strategies within the team, modulated by task complexity and team structure. For example, for highly interdependent tasks, proactive sharing of intentions and goals contributes more to team performance than sharing world knowledge (Singh et al., 2017). Further, high-performing teams employ implicit coordination strategies, such as proactive communication of future goals, which improves performance in complex tasks but relies on shared mental models and accurate mentalizing (Butchibabu et al., 2016). Thus, understanding the interplay of these mechanisms is essential for modeling collaborative behavior and designing agents capable of human-like teamwork. Recent research has also explored fluid team structures in dynamic settings. For instance, self-organizing teams, particularly in crisis management, demonstrate flexible, adaptive role allocation but also face risks of role ambiguity negatively impacting performance (Jobidon et al., 2017). Studies on ad-hoc teamwork (Stone et al., 2010; Mirsky et al., 2022) extend these ideas into the human-agent collaboration domain, underscoring the challenges of coordinating with unfamiliar partners without prior agreement. We aim to investigate and model the (cognitive) coordination and dynamics that make the process of self-organizing in teams possible and increase their performance and efficiency. Fluid collaboration, as we define it here, relates to the optimal mode of operations within self-organizing teams when flexibility leads to the best outcomes.

### 2.2 Human-agent collaboration

The theory of teaming has been applied to human-agent collaboration, with a number of expectations and promises (Sukthankar et al., 2012; Zhang et al., 2021) but also many open challenges (Natarajan et al., 2023). Related works investigate core concepts of teamwork with intelligent agents, such as team cohesion (Lakhmani et al., 2022) or the influence of interdependence (Verhagen et al., 2022). Recent surveys conclude that the field is heterogeneous, lacks clear definitions of key concepts, and uses terms like human-agent collaboration, human-agent teams, or human-autonomy teaming (HAT) often interchangeably (Berretta et al., 2023; O’Neill et al., 2022).

The notion of FC proposed here is related to concepts such as self-organizing and ad-hoc teams, which increasingly are being explored with human-agent teams (Tabrez et al., 2020; Natarajan et al., 2023). In human-robot teaming, concepts such as team fluency (Hoffman and Breazeal, 2007; Hoffman, 2019) and cross-training (Nikolaidis and Shah, 2013) have been proposed to improve joint performance by fostering anticipatory behavior and mutual adaptation. However, achieving human-like flexibility in coordination remains an open challenge, especially in unstructured or novel environments where dynamic mentalizing–the ability to flexibly model partners’ intentions–becomes critical (Bendell et al., 2024). Agent abilities such as anticipating partners’ needs and subsequent proactive behavior (such as information pushing) are being explored but are not fully developed yet (McNeese et al., 2018).

Task-oriented collaboration has been traditionally discussed as being inherently connected to the process of planning together (Grosz and Hunsberger, 2006), which has been put forth as one of the basic necessary principles to model collaboration between humans and artificial agents (Cila, 2022). Part of this planning process is to assign roles, which is found to improve collaboration as it raises the awareness towards responsibilities of oneself and other group members (De Wever and Strijbos, 2021). This process, however, requires an explicit exchange and negotiation of goals (Butchibabu et al., 2016) and is often too costly to be done on-the-fly or in time-critical situations (Unhelkar and Shah, 2016). At the same time, the explainability of agents in ad-hoc teams was found to improve team situational awareness, yet with reduced performance for experts (Paleja et al., 2021). Overall, these works focus on establishing cooperative structures (e.g., actions, plans, or roles) which then are not dynamically adjusted anymore.

The problem of action selection with others is commonly considered in the fields of Multi-Agent Planning (Torreño et al., 2017) and Multi-Agent Reinforcement Learning (Oroojlooy and Hajinezhad, 2023). The former bears the advantage of creating an explicit plan but requires domain knowledge to formulate a planning problem description; the latter is faster at inference time but requires much interactions in the environment for training (due to RL’s sample inefficiency), which is problematic in collaborative situations which exhibit a large variance and dynamics. Research on dynamic task scheduling and allocation in human-robot interaction has focused on the tasks, agent capabilities, and real-time performance monitoring (Alirezazadeh and Alexandre, 2022), cognitive load (Giele et al., 2015), and its application in real-world factories (Petzoldt et al., 2022). A real-time approach to task scheduling based on mutual trust has been presented by Wang et al. (2015). More recent work has started to use Large Language Models for coordinating tasks between multiple agents (Liu et al., 2024) and for incorporating Theory of Mind (ToM) into the behavior of agents (Li et al., 2023). Others (Andy et al., 2021) have underscored the significance of conventions in adapting to frequent new partners. Similarly, recent work by Lohrmann et al. (2024) highlights the relevance of identifiable and predictable robot behavior for human-robot collaboration in simple scenarios. Their PACT model is based on patterns that are optimized and then fixed over a collaboration, and was found to improve team dynamics and perception of the robot.

An increasing number of researchers employ synthetic environments for studying human-agent collaboration. Cooke et al. (2020) emphasize the use of such environments for investigating principles of cooperation (roles, responsibilities, or autonomy) and gathering insights about interactions even before developing an agent. Very prominent in research on collaboration are game scenarios, such as the computer game *Overcooked!* ^3^ in which players control virtual chefs in a simulated kitchen with the task to prepare ordered meals. Based on the original game, Bishop et al. (2020) proposed an evaluation testbed for human-agent interactions in which they developed several objective measures to evaluate the interaction in a Wizard-of-Oz scenario, also further describing possible studies using this method (Rosero et al., 2021).

In order to enable the development and evaluation of agents, different implementations of *Overcooked!*-like environments have been employed for research. The most prominent implementations are the overcooked-ai environment by Carroll et al. (2019), as well as gym-cooking by Wu et al. (2021). These implementations, however, simplify many aspects relevant for fluid collaboration. Most notably, agent movement is often grid-based or the time-critical aspect of the game is modeled in discrete steps. Further, these environments allow for control over the task complexity only through the kitchen layout, while the available recipes are predefined. Different benchmarks are based on or employ the domain, e.g., for multi-agent reinforcement-learning (Agapiou et al., 2022) or LLM-based collaborative agents (Sun et al., 2025). Several agent architectures were introduced for and evaluated within these environments, including approaches with hierarchical reinforcement learning (Aroca-Ouellette et al., 2023) and ad-hoc team partner adaptation (Carroll et al., 2019; Strouse et al., 2021). The environments are also used as testbeds for explicitly modeling Theory of Mind reasoning in collaboration.

### 2.3 Theory of Mind

Theory of Mind (ToM) is the capability of interpreting the behavior of others in terms of their mental states such as intentions, beliefs or emotions (Beaudoin et al., 2020; Wellman et al., 2001; Goldman, 2012). It encompasses drawing inferences on those mental states, predicting future actions of others, and integrating these predictions into one’s own social reasoning and planning processes. Therefore, ToM is a key cognitive skill required for everyday social interaction (Rivero-Contreras et al., 2023). In collaborative settings and teams, ToM plays a crucial role in forming and updating shared representations that underlie anticipatory and effective coordination of action (Krych-Appelbaum et al., 2007), especially in dynamic or ambiguous environments (Scheutz et al., 2017; Bendell et al., 2024).

Different accounts have been put forward in Psychology and Philosophy to explain ToM. Most notably, Theory Theory (TT) assumes that behavior and mental states are captured in terms of rules of folk psychology, while Simulation Theory (ST) assumes that the human mind simulates others’ perceptual or cognitive processes to infer mental states (Cruz and Gordon, 2003). Recent accounts argue for less dichotomous, combined approaches. Computational approaches to ToM mostly rely on formal representations like Bayesian modeling or epistemic logic ( 65% of approaches according to a systematic review by Rocha et al. (2023)). Bayesian Theory of Mind (BToM) deduces mental states by assuming rationality and running probabilistic inverse planning via the Bayes rule (Baker et al., 2011). There, computations of the evidence quickly become intractable in a complex environment such that, e.g., heuristics or simplifications are needed to make this approach applicable in real-life scenarios (Pöppel and Kopp, 2018), which also conforms with what has been found in human behavior (Bonawitz et al., 2014; Vul et al., 2014). For example, Gershman et al. (2016) showed that humans are more likely to assume habitual behaviors of others when they are under time pressure and under higher cognitive load, opting for simpler heuristics. Much research on collaborative agents focused on isolated inference tasks concerning specific mental states, like goal recognition. Chiari et al. (2024) have shown that inference time, accuracy and robustness of goal inference can be balanced by utilizing fast and slow models via meta-decisions to keep the fast solution or invest time for a potentially better one. Others have started to include contextual information into the inference problem. For example, Zhang et al. (2024) recently found that incorporating the timing of behavior or goal solvability can improve results towards human performances.

BToM also has been adopted to integrate mentalizing with other cognitive processes, like cooperation and communication (Stacy et al., 2024; Kahl and Kopp, 2015). Ho et al. (2022) argue for applying ToM as planning models for changing other mental states instead of just predicting them. Erdogan et al. (2024) argue for inferring higher-order abstractions, like trust, respect and affinity, via ToM through epistemic logic and abstraction rules. Yet, while there are diverse and interesting approaches to model ToM, most studies have employed “small worlds” scenarios, like the Sally-Anne false belief task, navigation (Pöppel, 2023; Stacy et al., 2024), or Hanabi (Montes et al., 2023). Few approaches have attempted to model ToM capabilities by way of machine learning, e.g., using supervised learning on synthetic data for which information about agent type or goal is available (Rabinowitz et al., 2018). With the rise of foundation models such as LLMs, their generalization of large-scale statistical data to model ToM is studied widely and has shown promising ToM capabilities for very specific and familiar scenarios, but poor generalization and robustness to variations (Gandhi et al., 2024; Shapira et al., 2024).

Notably, the modeling of ToM in and for collaboration has been widely studied in *Overcooked!*-like environments. Gao et al. (2020) describe an agent with human-like explanations via hierarchical models of the human user’s mind, which improved collaboration performance and perception of the artificial agent. Wu et al. (2021) propose Bayesian Delegation to infer the partner’s hidden mental states via BToM and integrates it into a multi-agent MDP for decentralized collaboration. Ying et al. (2024) use a two-level ToM with I-POMDPs to proactively decide when to request and share information via verbal communication. They formulate a planning problem to align joint plans of the team members and translate between their internal representation and natural language via LLMs. Participants rated this model as more communicative and helpful than other models.

In sum, while various aspects of Theory of Mind reasoning are increasingly being explored and integrated into artificial agents-particularly in collaborative settings-current approaches remain limited when it comes to collaboration on everyday tasks as outlined above. One reason for this is that current approaches are developed and tested in environments that do not pose realistic challenges, like demanding tasks under uncertain and changing conditions or temporal constraints. In contrast, existing models focus on isolated inference tasks or short-term action predictions rather than on the dynamics of shared mental models and the communicative mechanisms that could account for the real-time adaptation of commitments or responsibilities seen in robust, human-like collaborative behavior. This limitation becomes especially apparent in fast-paced, situated interactions where coordination must be anticipatory and continuously adjusted, i.e., in what we refer to here as fluid collaboration.

## 3 Defining and measuring fluid collaboration

With the goal to develop socially intelligent agents that can engage in FC with humans and other agents, we start by providing a definition of the specific kind of interaction we are targeting. Generally, we use the term collaboration to refer to any interaction in which two or more agents (humans or artificial) pursue a common goal by working together in a shared environment with interdependent (sub-)tasks. Mutual coordination of actions is hence indispensable between collaborating agents. An extreme case of collaboration is joint action in which the agents’ actions are strongly interdependent and closely coupled with each other (Sebanz et al., 2006), e.g., when carrying a table together. At the other end of the spectrum, the term cooperation has often been used to refer to situations in which agents are loosely coupled and carry out possibly separate tasks, but still need to support each other with regard to task interdependencies, e.g., by making sure to share resources and not to interfere with each other (Petzoldt et al., 2021; Petzoldt et al., 2022).

We focus here on collaborative interactions in special settings, namely, when the common goal needs to be pursued in a dynamic, uncertain or unpredictable environment. In such settings, a complete plan cannot be fully determined a priori and the collaborative activity cannot follow rigid patterns. Rather, the interaction partners have to engage in fluid collaboration, which we define to be characterized by frequent and manifold transitions of how the agents work together. More formally, we conceive of a situated collaborative activity as being composed of several constituents: a number of agents performing interdependent tasks that occupy resources. Tasks can be complex and comprise sub-tasks, with the bottom-most tasks relating to primitive actions executable in the environment. Resources can be tools that are used or entities that are consumed in a task or its corresponding actions.

We subsume both tasks and corresponding resources under the term collaboration units. In this view, successful collaboration requires the partners (1) to agree on an overall high-level task that achieves a common goal (e.g., preparing a salad), (2) to follow a suitable plan out of more basic, partially ordered sub-tasks that implement the high-level task, and (3) to implicitly or explicitly coordinate an assignment of units to individual agents who are then (temporarily) in charge of these units and the fulfillment of corresponding responsibilities. Agents can either assign themselves to a unit (self-assign), e.g., by picking up a resource or announcing verbally that one takes on a certain task, or they can try to assign a unit to another agent (other-assign), e.g., by asking or suggesting another agent to take care of a sub-task. This can imply a specific role of the respective agent and comes with the responsibility to execute actions related to the unit, e.g., the actions in a task or the actions that involve the resource. We call a unit which lies entirely with one agent assigned, whereas units which have more than one agent assigned to them are called shared. Crucially, the assignment established by the agents needs to comply with overall goal achievement (plan conformity) as well as the interdependency structure among the units.

**FIGURE 2:**
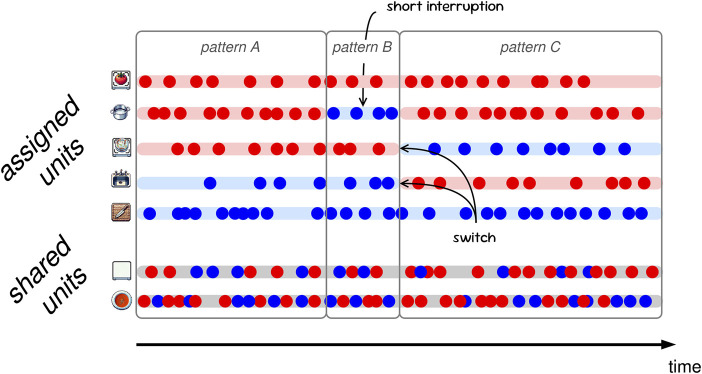
Illustration of dynamic collaboration patterns with different assignments of units to agents (Red and Blue) changing over time in a fluid collaboration. Dots represent actions by an agent on a unit (rows). Patterns refer to phases of temporarily stable, partial assignments. For example, pattern B differs from pattern A in that agent Blue has started to use the pot.

As mentioned before, and unlike in expert-based scenarios like surgery or military operations, where every agent is acutely assigned and aware of their task or role, collaboration in everyday scenarios is often ad-hoc and remains adaptive. That is, although the agents agree on and commit to the same overall goal, it is often not clear what task to collaborate on next or how to accomplish it. In our terms, the assignment of units is not set nor specified beforehand, but undergoes multiple and on-the-fly adaptations throughout the collaborative activity (see Figure 2 for an illustration). This is the defining feature of FC, and this fluidity is often necessary because tasks may arise unexpectedly, the execution of tasks may not be successful, the availability of resources may change, their assignment turns out to yield problems for commencing with tasks, or agents may self-assign collaboration units and hence start executing other tasks or using other resources. In other words, the above requirements for a functional assignment can become violated due to changes in the environment or the agents’ behavior. As a result, agents in FC are continuously faced with the need to monitor and evaluate the environment, the current assignment, and their performance on individual tasks and as a team–and to take coordinative actions if necessary. This way of collaborating is required to successfully cope with necessary (and constraining) hard interdependencies, while at the same time exploring and optimally leveraging soft interdependencies in dynamic environments (Johnson et al., 2020).

On the other hand, it is important to note that even FC requires phases of stability with suitable assignments persisting for a certain period of time. Such phases and how they change when agents adapt the assignment of units can be characterized as collaboration patterns that improve team performance and agent perception (Lohrmann et al., 2024). Patterns can be defined as temporarily stable assignments (Figure 2), i.e., partial or global mappings of units to their assigned agents, which tend to reoccur in a more or less generalized or specialized form. For the collaborating agents, such patterns can be useful. For example, they can scaffold how newly arising units are assigned and they provide a higher level of coordination and learning to the agents (e.g., when suggesting to prepare a dish in a similar manner as a previous one). FC, then, is characterized by agents’ behavior that brings about frequent changes of such collaboration patterns over time, along with the corresponding mechanisms to coordinate those. Importantly, these mechanisms may be internal and implicit, e.g., ToM reasoning for intention recognition or plan adaptation, or communicative and explicit, e.g., dedicated speech acts such as declarations, proposals, negotiation, grounding, or repair. We consider the fluidity of collaboration to result from exactly these adaptations.

### 3.1 Measures

Our definition of FC allows for formulating qualitative and quantitative measures to assess and analyze important characteristics of fluid human-human or human-agent collaboration. A framework for evaluating the impact of perceived and measured fluency (based on reaction and idle times) on the interaction was developed by Hoffman (2019). We want to complement this with metrics that operationalize key qualities of collaboration in general and FC more specifically. These measures can be used to analyze human-human and human-agent collaboration as described in the next section.

#### 3.1.1 Performance

Many researchers use measures of task performance as an indicator for successful collaboration. Under the assumption that environments and tasks are designed to require collaboration, this is a reasonable approach^4^. A performance score is typically modeled as a task success rate, possibly related to invested efforts, time, or costs.

#### 3.1.2 Intertwinement

One feature of the quality of collaboration may be equal participation and contribution by all partners. We thus define a metric that represents the deviation from an equally balanced contribution by all participants to all successfully executed tasks. For this, we need a set of tasks to fulfill in the environment T with given task structure s , e.g., subtasks ( ST ) s:T→ST×ST…×ST and a function a that indicates execution of a subtask by one participant a:ST→P . The intertwinement score it (Equation 1) is then defined as

$$it=\frac{1}{\left|T\right|}\underset{t\in T}{\sum }|P|\times \underset{p\in P}{\min}\left(\frac{1}{\left|s\left(t\right)\right|}\underset{st\in s\left(t\right)}{\sum }\delta_{a\left(st\right),p}\right)$$ (1)

where δ is the Kronecker delta, which returns 1 for the same values, 0 otherwise. The intertwinement score shows how much agents worked together at individual tasks, e.g., a 50–50 contribution to all subtasks of all tasks by two agents results in a score of 1. If every partner fulfills tasks alone, the it is 0. Similar to tasks, we can define the it for resources. There, T is the set of resources and ST is the single interaction with the resource.

#### 3.1.3 Fluidity of unit assignment

We define the fluidity of a collaboration unit u in terms of the transitions of assignments to different partners that interact with it. Let $S_{u}=\left(p_{1},p_{1},p_{2},p_{1},\ldots \;\right)$ be the sequence of actions by partner $p_{i}$ on unit u with length n . The fluidity of a unit assignment (Equation 2) is given by the relative frequency of transitions in the sequence of actions

$$F_{u}=\frac{T_{u}}{n-1}$$ (2)

where $T_{u}$ is the number of transitions of assignments of unit u . A transition occurs if $S_{u,i}\neq S_{u,i+1}$ .

#### 3.1.4 Pattern dynamics

Assume a set of units U , a set of collaboration patterns Φ , and a sequence of actions S . Let f:Φ→P(U) be a mapping from a pattern to the set of units assigned in this pattern, without information to which partner the unit is assigned to ( P is the power set). Building on the previously defined fluidity score, we define a pattern dynamics score (Equation 3) as the minimum, across all patterns, of the mean fluidity of units within each pattern.

$$pd=\underset{\phi \in \Phi }{\min}\left(\frac{1}{\left|f\left(\phi \right)\right|}\underset{u\in f\left(\phi \right)}{\sum }F_{u}\right)$$ (3)

This score reflects the extent to which partners followed or deviated from a single collaboration pattern in the considered sequence of actions. Note that this score depends on the units and patterns considered. For example, a simple set of patterns would correspond to considering resource and task units. More fine-grained but perhaps less consistent patterns are possible when considering, e.g., area resources, special kinds of equipment, machines, or finer subtasks as units that are assigned.

Clearly, when trying the measure the kind or quality of a collaborative interaction, and especially FC, communication between the partners is another highly relevant feature. Several metrics have been proposed in Linguistics to measure the use of situated language or multimodal behavior in dialogue or conversational interaction (Walker et al., 1997; Fusaroli and Tylén, 2016; Louwerse et al., 2012; Kopp, 2010). These measures are naturally important in FC, but outside the scope of the present paper.

## 4 Studying fluid collaboration

### 4.1 Scenario: Cooperative Cuisine

What environments or settings are suitable for studying fluid collaboration? Above, we have referred to several real-world scenarios that solicit and even require FC (e.g., preparing meals or cleaning a room together). However, for the scientific study of FC between humans and artificial agents, and with a particular interest in analyzing the role of ToM reasoning in these tasks, we require a task environment that fulfills a number of requirements: (1) The tasks to be carried out need to align with the competency of humans, but must require multiple agents to work together. Further, the tasks must be reproducible, with a complexity that is controllable and configurable. (2) The environment must naturally allow for human-human collaboration but also for integrating artificial agents (at any number) in these interactions. Their potential action space should be on par with that of humans. (3) The environment should allow for imposing scalable demands such as time pressure or multiple tasks. It should foster ad-hoc collaboration without necessary long discussions or explicit task separation prior to acting. (4) Agents should be able to act at any time, concurrently, and with continuous control over their actions, unlike in turn-based games/environments. (5) The environment must allow partners to communicate with each other naturally, e.g., via spoken language, which can be captured and possibly even manipulated by study designers. (6) Finally, in addition to capturing audio or video data of human participants, the environment must enable the time-sensitive recording of all events and actions for later fine-grained analysis.

**FIGURE 3:**
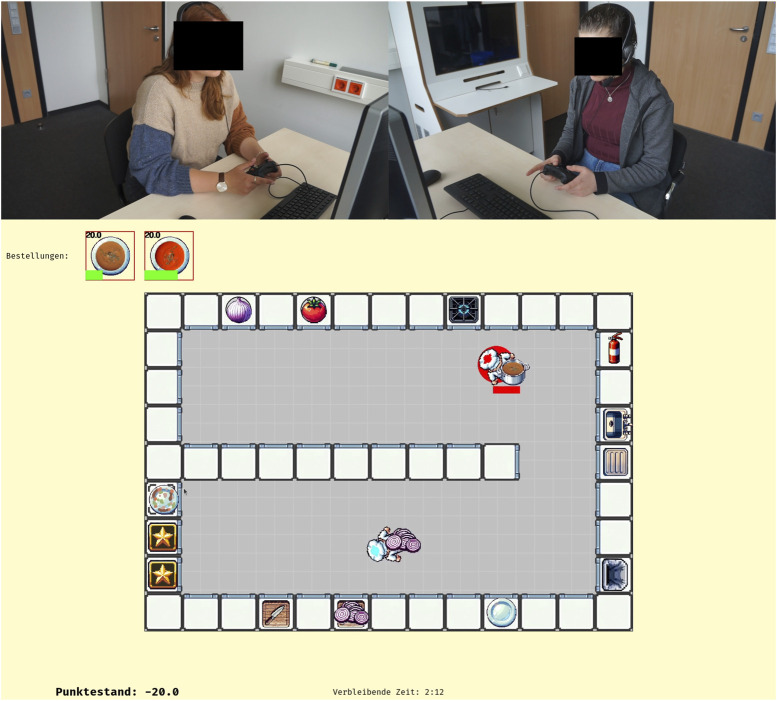
The Cooperative Cuisine main screen. Partner A and B are collaborating to complete the orders shown at the top left.

To develop such an environment, we opted to build on the *Overcooked!* game scenario, a kitchen task simulation which has been widely adopted in research on modeling machine ToM abilities (Wu et al., 2021; Bishop et al., 2020; Gao et al., 2020). Yet, to meet the above-mentioned requirements and to overcome limitations of current approaches, we have improved upon existing implementations by building the Cooperative Cuisine (CoCu) environment for exploring fast-paced, real-time collaboration between an arbitrary number of humans and artificial agents in a simulated kitchen setting (see Figure 3). The overall task for the participants is to jointly prepare meals according to incoming orders. For this, they have to fetch, process, and combine ingredients according to given recipes and using available resources (objects, appliances). Demands on the agents are manifold and finely controllable: Orders come in at a given pace and they vanish when not being served within a limited period of time (punished with a negative score), orders require specific tasks and resources which can be limited and require fixed amounts of minimal or maximal time themselves (e.g., chopping lettuce or cooking soup, but also catching fire when cooking too long), the kitchen layout (scenario) can be designed to specifically afford or restrict access to certain tasks or resources for single players. Cooperative behavior is hence required as participants need to work on tasks and employ resources in highly coordinated ways. Crucially, the environmental dynamics require a high degree of flexible adaptation and continuous adjustment for agents to be successful.

A distinguishing feature compared to existing similar environments (cf. Carroll et al., 2019; Wu et al., 2021; Liu et al., 2024; Agapiou et al., 2022) is the possibility of continuous movement and hence less restricted actions. While it improves the experience of human participants, the higher complexity poses new challenges during the development of autonomous agents that require new approaches to deal with it (such as navigation, movement, or symbolic actions). The continuous movement also enables research on the combination of natural language communication with analogous signaling through environment action [see e.g., Royka et al. (2022)]. Humans are experts in extracting social signals from simple movements (Heider and Simmel, 1944). Enabling such fine-grained actions hence is important to allow seamless situated coordination in simulation environments.

Also in contrast to other environments, CoCu is designed with human studies in mind, featuring guidance through the study procedure with a tutorial, instructions, and résumé screens. At the same time, artificial agents can easily be deployed and act as full participants. Technically, we have implemented CoCu as a distributed, extensible game environment. Participants (human clients and artificial agents) connect via the same Websocket interface for continuously requesting the environment state and updating actions. Inter-player communication is done via an external voice chat server, external to but synchronized with the game server. Most aspects of the environment are highly configurable by adjusting variables such as the kitchen layout and task complexity (among many others). Current implementations provide a fixed set of recipes and objects, ranging from a single type of ingredient (Carroll et al., 2019) to three ingredients, fire mechanic, and trashcan usage (Liu et al., 2024). In CoCu, the item and counter types and the recipes are defined in configuration files. Therefore, their number is not limited and can be extended to the necessary complexity. This allows for a large variety of controlled experimental settings. Further, increased task complexity positively influences team trust and process satisfaction (Nah et al., 2011), motivating that the adaptable complexity in CoCu could potentially enhance team collaboration and satisfaction in virtual environments. CoCu also features extensive functionality to monitor and record the interactions within the environment, also including filtering for specific events to aid detailed analyses of the interactions. Our implementation in Python^5^ allows for direct interfacing with other existing approaches.

### 4.2 Study: fluid human collaboration in Cooperative Cuisine

We have conducted a study to see how humans collaborate in the CoCu environment and to which degree they show aspects of FC. More specifically, we wanted to carry out a fine-grained analysis of their behaviors and communication in order to identify and validate key aspects of FC as defined above. Furthermore, by investigating ToM processes in how humans naturally adapt to and infer each other’s intentions in a dynamic, fast-paced collaborative setting, we aim at enabling the development of FC-capable artificial agents.

#### 4.2.1 Design

**FIGURE 4:**
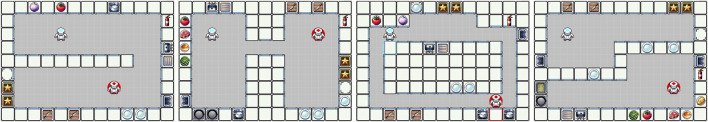
The four scenarios with specific kitchen layouts used in the study (1–4, from left to right).

Four different scenarios were designed, each of which meant to focus on some especially interesting aspects of FC. Figure 4 shows the corresponding kitchen layouts used in the different scenarios. The first scenario was designed to see if, when, and how the assignments of collaboration units are organized and change over time. For example, participants could assign separate tasks based on the meal (one partner could prepare tomato soups and the other could prepare onion soups), or they could assign separate resources (one player could work in the top half of the kitchen and the other on the bottom, using the middle counter row for handing over objects). Resource separation is more effective because of the long travel between the top and bottom parts of the kitchen.

Scenario two comes with a more complex recipe (Burger) and a prominent but small gap that requires low-level coordination when navigating the kitchen. The relevant counters are positioned so that exchange from one side to the other is not possible and for the completion of meals participants have to pass the gap quite often.

The third scenario restricts participants’ movements to a long circular corridor, where they cannot move past each other. The challenge is to coordinate movement for tasks or resources so that the partner is not blocked. The most time-consuming interaction is the chopping of ingredients. To perform well, participants hence have to coordinate who works at which cutting board and how they can fluidly put ingredients on/off the board.

Scenario four separates the participants into two-halves of the kitchen, each with specific resources. Thus, they are forced to collaborate by handing over objects via the middle counter. With this, we wanted to investigate whether participants develop a certain collaborative strategy or whether their actions are primarily driven by the situation with its current requirements and affordances. Further, we wanted to see from their communication whether participants also attend to the other half of the kitchen where they are not placed, e.g., for anticipatory coordination. With the four scenarios, we cover the whole range from soft interdependencies (collaboration opportunities) to hard interdependencies (environment constraints, limited access) (Johnson et al., 2020).

#### 4.2.2 Procedure

Two computer setups were set up in separate rooms at our university. 30 participants in N = 15 dyads (Age: M=28.33 , SD=6.76 ; Gender: female=15 , male=13 , other=2 ) played through all four scenarios of CoCu together (see Figure 3). They controlled their in-game characters using game controllers and could freely talk to each other via a headset connection. All communication and the computer screens were recorded. In addition, the participants were filmed during the interactions. All relevant information about in-game events was logged for later analysis.

Upon arriving at the lab, participants received a short introduction. After being seated at the computer, they could then individually explore the environment in a tutorial with information about controls and game mechanics displayed. Participants could decide themselves (by button press) when to conclude the tutorial and proceed with the study. In an instruction phase before each scenario, possible recipes to prepare were displayed. The game started when both participants confirmed to proceed to the actual scenario. They had 5 min in each scenario to prepare meals and complete orders. After the scenario ended, participants were given a résumé of their performance (including served meals and the final score). Finally, a post hoc questionnaire was administered, containing questions about demographic data as well as questions asking participants’ ToM abilities such as inferring their partner’s future actions and needs on a 7-item-Likert-Scale (consisting of questions like “I could predict my partner’s next actions well.”) with scores ranging from −3 to 3 points.

#### 4.2.3 Analysis and results

**FIGURE 5:**
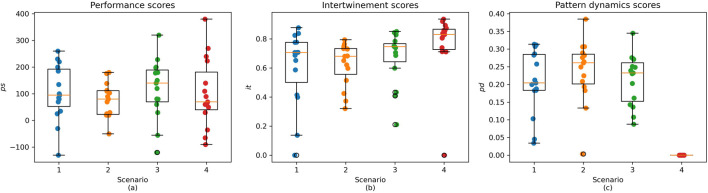
Performance scores (left), intertwinement scores (center) and pattern dynamics scores (right) in scenarios 1–4.

To analyze the interaction data, we adopted both a quantitative and qualitative approach. Quantitatively, we applied the above-defined performance, intertwinement, and pattern dynamics scores to the game logs. Figure 5 shows mean values for all four scenarios. The results indicate differences between scenarios as well as dyads. Reasons for that were how well participants managed to control their chef (experience with controller controls), strategies, familiarity with the domain, and familiarity with the collaboration partner. In general, all dyads were able to prepare and serve meals and fulfill tasks. The mean intertwinement score over all scenarios is above two-thirds, showing that participants worked together on single tasks and that CoCu encourages task-level coordination. The pattern dynamics scores were calculated with two patterns: separation based on task units (e.g., all actions corresponding to the preparation of a single meal) and separation based on resource units (here, all counters except free counters used for passing objects). The pattern dynamics scores over the first three scenarios show that the dyads, on average, did not follow only a single pattern.

However, the data also indicate that the pattern dynamics score is not a reliable predictor of performance outcome. For instance, in Scenario 1, both D14 ( ps=25 ) and D04 ( ps=85 ) exhibited low pattern dynamics scores ( pd=0.03 and pd=0.05 , respectively) yet performed comparable to D03 ( ps=35 ) and D01 ( ps=95 ), which had high pattern dynamics scores (0.28 and 0.29). This suggests that the correct pattern change is crucial for achieving high performance. Still, it is noteworthy that the pattern dynamics scores of the five dyads with the highest performance scores in Scenario 1 ranged from 0.18 to 0.31, placing them in the upper half of the pattern dynamics score range among all participants, with performance scores exceeding 180.

At the same time, some values are close to zero, indicating that individual dyads sometimes relied strongly on single patterns. Scenario four stands out in this regard. As expected from the scenario’s design, a high intertwinement score indicates particularly strong collaboration, while the low pattern dynamics score proves that interactions followed a consistent pattern because participants could only interact with the resources in their separated work areas.

**FIGURE 6:**
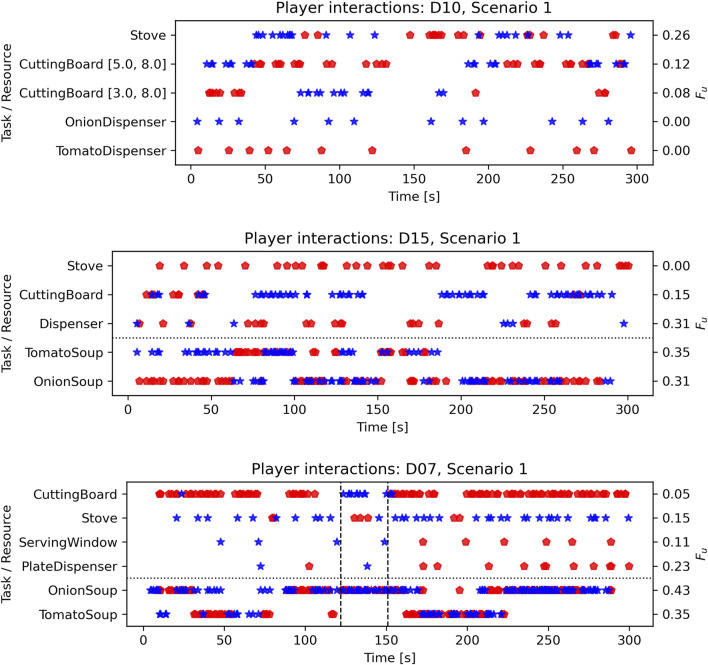
Assignments of units (resources with corresponding tasks) to the players (red and blue) over the course of three selected dyadic interactions in Scenario 1. The selected dyads have similar pattern dynamics scores in Scenario 1: $pd_{\mathrm{D10}}=0.204;\;pd_{\mathrm{D15}}=0.188;\;pd_{\mathrm{D07}}=0.183$ .

To shed more light on the collaborative interactions and possible behavioral patterns that might characterize FC, we qualitatively analyze interesting situations observed in the study. For *Scenario 1*, we focus on three of the 15 dyads (D). Figure 6 shows how different collaboration units (resources and corresponding tasks) are assigned to or assumed by the different players and how these assignments change over time. D10 is one of the few dyads that did not use the middle counter, making their strategy less optimal. Remarkably, the ingredient dispensers are assigned to (i.e., used by) different players throughout the interaction ( $F_{u}=0.0$ ), while the two cutting boards and the stove have varying assignments and were used by either player as needed. One can also see cases of shared assignments of the stove and a cutting board, indicated by a concurrent use (red and blue marks overlap). At a higher level, this collaboration displays the deployment of largely stable patterns that were not changed during the interaction (e.g., $F_{u}$ values close to 0 for each cutting board: 0.12 and 0.08).

In D15, a shift from task to resource assignment occurred. Participants start by splitting the tasks (different meals), but after some time, they switch to a strategy in which they split the resources instead of the tasks. While the stove is used by Red exclusively, they initially share the cutting boards. Around the 60-second mark, however, a switch happens when the players realize that orders are time-limited and they cannot complete the order with their current strategy. Their solution is that Blue assumes the assignment of cutting ingredients, while Red provides the ingredients. This is coordinated verbally. Calculating additional $F_{u}$ scores before and after the switch highlight the change from assigned task units before the switch (tomato and onion soup $F_{u}=0$ , indicating assignments) to shared units after the switch ( $F_{OnionSoup,>60s}=0.41;F_{TomatoSoup,>60s}=0.39$ , indicating shared units). Conversely, the resource units change from shared to assigned units ( $F_{Dispenser,\leq 60s}=1.0;F_{CuttingBoard,\leq 60s}=0.54;F_{Dispenser,>60s}=0.2;F_{CuttingBoard,>60s}=0.07$ ).

D07 already starts with a separation based on resources, but the blue player responsible for acting in the top part of the kitchen also serves at the bottom. Interestingly, this pattern is interrupted at around the second 120. While Blue is responsible for serving meals after preparing them on the stove, Blue starts to use the cutting board and Red uses the stove instead. This pattern holds only for a short period of time. After 30 s, they re-established the original assignment pattern with the optimization that Red served the meals and picked dirty plates from the plate dispenser. This change was triggered by Blue’s failure to serve a meal and stay longer in the bottom part of the kitchen than usual. The players quickly adapted their behavior after an initial assessment and short verbal discussion of this time-critical failure. However, the following pattern changes were not communicated explicitly and emerged without further explicit coordination. This resulted in the need for Red to break the assignment and move to the top part. Here, Blue is required to mentalize about Red, who breaks the arrangement by moving to the top and grabbing onions from the dispenser. Blue “acknowledges” the change by using the cutting board.

**FIGURE 7:**
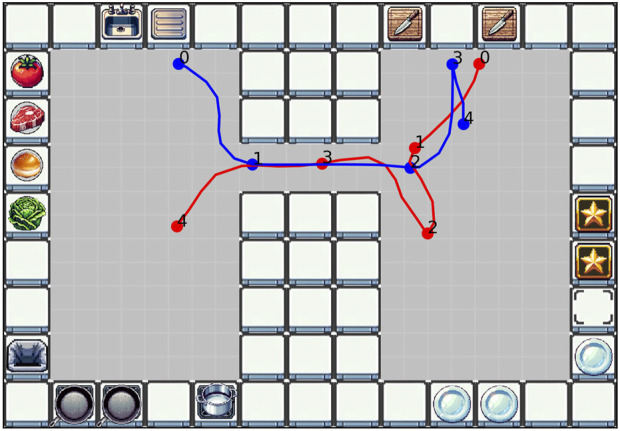
Two players coordinating when passing through the bottleneck in Scenario 2 (D02).

In *Scenario 2*, coordination is required when both players want to pass the corridor simultaneously. Figure 7 shows a situation where both players want to pass the corridor. Blue passes straight through, while Red, without pause, moves to the side instead of entering. This all happened without verbal communication between the players, and both players must understand what the other plans to do next. This could be a shallow movement heuristic, a deep prediction of intents, or a combination of both. Red moved to the bottom side of the corridor instead of up, which could be because Blue carried a steak that needed to be cut, with the path to the cutting boards being blocked if Red moved to the top. The space to move through can be seen as a collaborative unit that must also be allocated and coordinated. The described situation happens within 2 s, highlighting that this implicit coordination happens fast.

**FIGURE 8:**
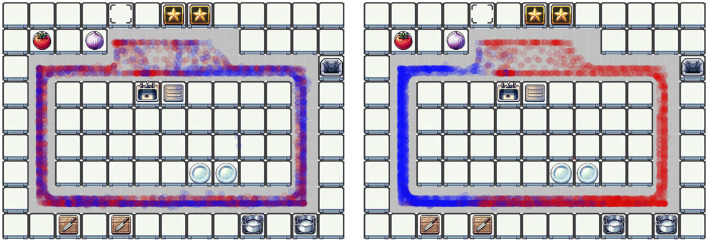
Overall location of players in Scenario 3 (left: dyad D05 with $pd_{\mathrm{D05}}=0.345$ ; right: dyad D07 with $pd_{\mathrm{D07}}=0.137$ ).

*Scenario 3* reveals different ways in which strategies can emerge. One way is the active negotiation of a strategy. In D05, after a short time of exploring the environment, the participants proposed their strategy of always moving counterclockwise through the kitchen to avoid blocking the paths for each other. This worked sufficiently well, and they stuck to this general pattern for the whole duration of the interaction. As a result, both players got to work almost at every location in the kitchen (Figure 8, left). Another way is an adaptive evolution of a pattern. This is seen in D07 (Figure 8, right), where the participants did not explicitly discuss a strategy. Instead, they started working on preparing the ingredients for the meals as needed. As a result, they split their tasks spatially based on the kitchen topology, with blue keeping to the left and red keeping to the right. In addition, this separation is also reflected in the pattern dynamics score; dyad D05 with higher $pd_{\mathrm{D05}}=0.345$ (on the overall higher end) and dyad D07 with $pd_{\mathrm{D07}}=0.137$ (which lies on the overall lower end). Interestingly, both dyads achieved a comparable performance score (120 and 140, respectively).

In *Scenario 4*, the blue player of D01 tried to assemble a burger using a raw burger patty, not noticing that it must be cooked. Red immediately noticed this mistake and intervened by correcting Blue verbally. In this case, Red had to mentalize and infer the immediate goal of Blue as “making a burger”, even though Blue executed the processing steps in a wrong order. Interactions like this require mentalizing based on close monitoring of the partner’s actions. Further, when one or multiple hypotheses about the partner’s intention are formed, they are checked bottom-up against the observed actions or verbal utterances. Here, the observed behavior does deviate from the top-down hypothesis and the resulting conclusion is not to change the goal hypothesis, but to attribute to the other agent a lack of knowledge and to trigger a corresponding corrective action.

Insights from the post hoc questionnaire show that people state that they were able to anticipate their partners’ moves ( M=1.74,SD=0.59 ) and, likewise, thought their partners could anticipate them ( M=1.32,SD=1.29 ).

### 4.3 Discussion

Although our analyses of the gathered interaction data are far from being complete, they already reveal many interesting insights. First, the definition of FC put forward in Sections 3.1 provides a useful framework for analyzing human collaboration in CoCu. By applying concepts like collaboration units (tasks, resources), assignments, and patterns of those, we find many interesting instances of how humans organize and coordinate their collaborative activity in real-time and on the fly. Perhaps most interestingly, all interactions in CoCu exhibit phases in which more or less stable patterns and assignments are established and acted upon. This did not require detailed planning nor lengthy explanations during the time-constrained tasks (albeit discussions were more in-depth between the actual task execution phases). Instead, those assignment patterns emerged over time and resulted from adaptations to task needs and environmental constraints. At the same time, such patterns are not exhaustive (they do not fixate use of every resource or task) nor persistent (they hold for limited periods of time), as partners dynamically and continuously adjust them to changing circumstances in order to maintain and optimize task performance. This happens with remarkably little explicit communication. Rather, human players used brief, efficient exchanges to communicate intentions, update others on task status, or offer clarifications. In scenarios where participants have to deal with limited resources (e.g., narrow corridors), near-instantaneous coordination of low-level actions such as direct movements was evident. Implicit signaling through non-verbal cues and movement patterns apparently was sufficient for participants to “read” each other’s intentions and avoid conflicts without explicit negotiation. Likewise, participants often corrected each other in real-time when errors or suboptimal actions were detected. This reveals a monitoring of each other’s behavior and, further, an assessment not only of the intentions but also the knowledge or skill of the other. Human participants in CoCu hence demonstrate a highly dynamic and efficient process of Theory of Mind, quickly adapting to others’ intentions inferred from their movements, assumed task assignments, or short verbal exchanges.

Indeed, according to the post hoc questionnaires, participants state that they and their team partner applied successful mentalizing during their collaboration. However, the self-reported post-hoc mentalizing ratings cannot provide a meaningful insight into the ongoing mentalizing process during the interaction. However, intermitting answering of questionnaires would have had a biasing influence [cf. Wildman et al. (2014)] in itself. One aspect that should shed light on the participants’ ongoing ToM reasoning is their communication behavior, playing a critical role in most team settings and for team performance (Mesmer-Magnus and DeChurch, 2009). An analysis of the communication within the interactions is ongoing and will very likely reveal important insights in coordination strategies and team processes.

An important conclusion is that FC in the Cooperative Cuisine environment requires specific, highly dynamic forms of mentalizing (ToM). Participants seem to combine fast and “good-enough” mentalizing with task-based perception, planning, action, and communication processes. On the one hand, collaboration partners need to react to and reason about new events and coordinate their interactions with each other quickly. This requires fast and reactive planning of one’s own actions and awareness of the actions of team partners in the environment. We see that participants take into account other agent’s intentions, beliefs, or skills in their planning. This crucially includes intentions or beliefs about the current assignments and collaboration patterns, both of which are subject to continuous coordination in FC, such that another agent’s needs or attempts to adjust these assignments need to be inferred and responded to on the fly. On the other hand, finding a complete shared plan prior to acting is impossible in CoCu due to the dynamic and unpredictable environment. Replanning and sharing complete plans is also too costly (cognitively and communicatively) and would compromise performance. Fluid collaboration in CoCu hence hinges on the agents’ ability for continuous coordination, which in turn is rooted in fast and efficient mentalizing (ToM reasoning) and combining it with one’s own actions seamlessly.

## 5 Modelling fluid human-agent collaboration

Our conceptual and empirical results provide a solid basis for moving toward artificial agents that are able to collaborate with other agents (including humans) in the Cooperative Cuisine environment. Considering the state of the art in the field, we note that the main challenge in this involves developing machine ToM abilities that allow for dynamic mentalizing in conjunction with action planning and communication.

In order to develop a computational ToM framework suitable for dynamic mentalizing, we start by looking at human ToM processes in similar domains. To that end, we subscribe (and contribute) to the growing field in Psychology, Cognitive Science, and Computer Science, that investigates ToM from the point of view of situated, resource-bounded cognition. A growing body of research has started to show that general cognitive principles, such as bounded rationality, heuristics with corresponding cognitive biases, or dual-process accounts that combine fast associative (system 1) and slower deliberative (system 2) thinking, also apply to ToM reasoning (Pöppel et al., 2021). This is important for two reasons. First, the solutions that human cognition came up with, to reconcile complex, uncertain social reasoning with real-time requirements in interactive settings, can provide hints for computational modeling approaches. Second, since our AI agents shall collaborate with humans, it will be important for them to take into account the ways in which humans deviate from optimal reasoning and behavior.

A second important consideration is that, in developing FC-capable agents, concepts of robust and efficient ToM need to be taken substantially further, as we are not modeling a passive observer only, but an active collaboration partner whose mentalizing leads to actions that in turn influence the other agent mentalized about. This bears important implications. One is that mentalizing and action planning need to be integrated in intricate and closely coupled ways. An agent’s mentalizing will, on the one hand, be focused and narrowed down by its current goals and action intentions (e.g., to confirm whether the other agent complies with a current assignment). On the other hand, real-time monitoring and planning will inevitably yield partial plans and open contingencies, which pose important queries for mentalizing or communication (e.g., to determine the necessary basis for one’s own decisions). In addition, cognitive resources must be shared between mental processes involved in mentalizing, planning, and communication. Another important implication is that collaboration unfolds in a social interaction and feedback loop (Kahl and Kopp, 2023), in which one agent’s beliefs about the other lead to actions that will impact the other agent’s mentalizing, which in turn lead to (re-)actions that will again require adapted mentalizing in the first one.

**FIGURE 9:**
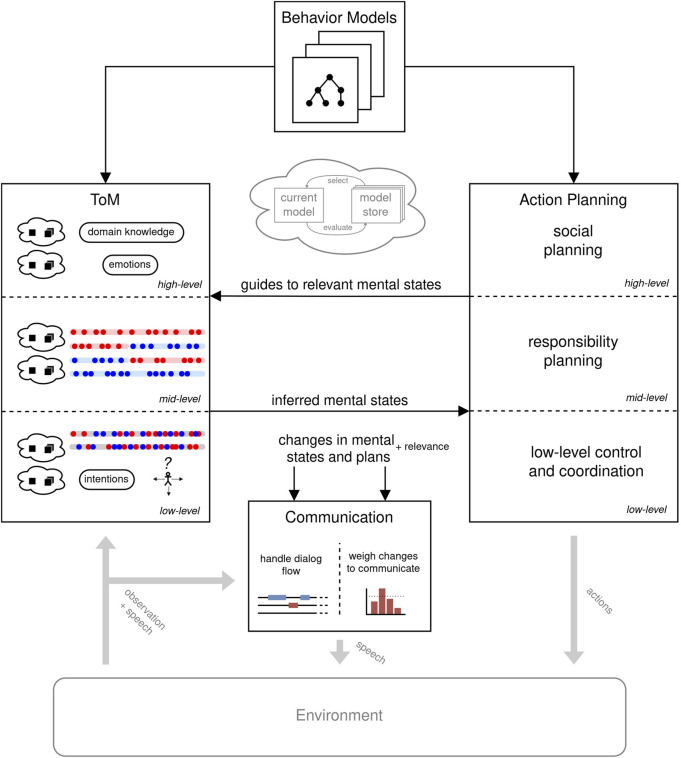
Overview of a proposed architecture for an FC-capable agent based on dynamic mentalizing through efficient and action-driven ToM.

In the following, we tackle foundational steps in this direction. We base our approach on our definition of FC applied to the Cooperative Cuisine scenario, according to which we distinguish different levels of collaborative behavior with corresponding mentalizing and planning processes (see Figure 9). Each level is characterized by how much “deep thinking” with corresponding computational resources is required. Higher levels correspond to an explicit, heavy-weight ToM, which is computationally costly but less often required. These inferences concern, e.g., another agent’s general domain knowledge, stance, preferences, or emotions, which are less time-critical and less relevant for most action changes. We hence assume heuristics to be applied at this level (e.g., assuming that the other agent is generally cooperative or has knowledge and skills like mine). At a mid-level, beliefs or intentions about assignments and patterns are dealt with. Such ToM inferences are crucial for understanding and responding to situational changes in the environment or optimizations and, therefore, occur more frequently. The latter can be hinted at directly by deviating from patterns or unexpected actions, in which case the required ToM reasoning to infer new mental states can be granted more computational resources. At this level, the agent needs to plan for who is responsible for which unit, coordinating shared units, or performing actions related to assigned units. Here, a planning algorithm can be employed to build a hierarchical plan structure that is shaped by prior knowledge but leaves room for adapting to situational changes. We note, however, that also at this level there are no longer-term plans being formed during fast collaboration. Indeed, we see in our data that humans defer optimizations and explicit coordination to directly before the actual collaborative interaction starts. At the lower level, mentalizing about action-related states (e.g., distal or motor intentions) is important for low-level FC such as coordinating movement and tight interaction on shared units.

### 5.1 Resource-rational mentalizing

As argued before, ToM computations in FC must be adaptive to the currently available resources to achieve the best inference under the current restrictions. In previous work, we have shown that understanding the mental state of another agent can be greatly simplified and made more efficient by drawing on assumptions about that agent. For instance, when we presume that an agent is knowledgeable and has correct beliefs concerning the world and the consequences of actions, one can refine mentalizing to intention recognition. Conversely, mentalizing without such assumptions becomes more intricate as one must consider intentions in relation to potentially incorrect or incomplete beliefs. These assumptions can be straightforwardly formalized within a Bayesian ToM (BToM) framework, corresponding to different causal agent models or clamping single variable to specific values.

We have introduced a computational model addressing this in earlier work (Pöppel and Kopp, 2018; Pöppel, 2023). We explored how the trade-off between accuracy and complexity can be resolved through what we term satisficing mentalizing (similar to Simon’s concept of satisficing decision-making (Simon, 1956)). This approach involves dynamically switching among BToM models, each related to certain simplifying assumptions and incurring different computational costs. These models can also mimic cognitive biases observed in human mentalizing. Model-switching is governed by evaluating models using a surprise measure that estimates their expected accuracy and weighing it with their expected costs. Thus, mentalizing is always performed using the simplest, most efficient model as long as it sufficiently predicts the observed agent’s behavior. This method was implemented and validated with real human behavioral data from a navigation task, where human observers also exhibited egocentric biases (Ross and Sicoly, 1979). The results indicated the superior performance of the model-switching approach over a single-model strategy, both in terms of efficiency and accuracy (Pöppel and Kopp, 2018).

In FC, switching between situation-specific assumptions is crucial. On the one hand, we have different mental states to infer, with different temporal resolutions in expected changes. On the other hand, the agent can encounter a wide range of partner types in different scenarios. To enable satisficing mentalizing in FC, we propose to use different methods to infer mental states in parallel. Those models must be able to forecast how accurate and resource-hungry they are in a given situation. Therefore, which methods to use for satisficing mentalizing is currently not restricted as long as a conversion between the employed representations and ontologies exists. Those can be formal representations (for BToM or epistemic logic) or text (for LLMs) (Rocha et al., 2023). A higher-level “meta-cognitive” control can check if the current belief is still probable or reject a model otherwise, giving computationally demanding methods more time to infer a new belief. The better the models reflect the other agent’s mental processes, the better the switching and inferencing will fit the situation. Further, the theory can apply when the set of available models is extended, e.g., through adapting current models or inducing new ones during the interaction. Even if these new models are not perfect, due to the switching strategy, the models can take effect in fluid changes when their bias or assumption applies.

### 5.2 Action-driven mentalizing

We have integrated the aforementioned approach to satisficing mentalizing into our FC agent framework and enhanced it with a mechanism to further refine mentalizing dynamically based on the cooperative agent’s action intentions (Schröder and Kopp, 2024). Being an actor in the environment itself, the mentalizing agent will have at least one action model that it applies for its action planning. At best, it has several models that can be applied in different scenarios, similar to the satisficing approach to ToM. Utilizing these action models also in ToM has several benefits. First, ToM outputs can be better applied to action planning if both already use the same semantics, syntax, and variables. Second, adapting the model, e.g., for better planning, would automatically extend the capabilities of ToM. Third, it would make communicating own actions and beliefs easier when both rely on the same assumptions. The inference methods used in the ToM models need to harness the action planning models. For example, BToM has been used to exploit the dependency between actions and intentions (Wu et al., 2021). Here, adapted planning models change the different BToM models by changing the action-intention probability distribution.

Another main advantage of coupling ToM reasoning with action planning is that the inference process can be sped up by focusing on mental states of the other agent that are assumed to be critical for one’s own current action planning. Allowing the planning component to “request” which mental states to infer is, therefore, beneficial for the efficient usage of cognitive resources. Furthermore, this form of action-driven mentalizing will help to prioritize which features to consider about the mental states, e.g., by answering “is she more likely to move towards tomatoes or onions?” the agent can largely ignore movements towards a different location. Applying these restrictions in the Bayesian inference process directly reduces computational overhead by omitting variables and values.

Taken together, these approaches must be integrated in a joint architecture for an artificial agent in FC. Figure 9 depicts a proposed architecture. It integrates the sets of planning models with the Mentalizing and Action Planning components. Both are hierarchically structured with a priority of lower-level execution. The higher levels are computed less often but then with more resources. The rest of the time, they are in a “monitoring state” where they check for deviations from the expected behavior, which would justify computationally expensive thinking. The action models can configure the ToM computation using the action-driven focus of ToM described above. The model-switching approach of satisficing mentalizing is applied in the ToM component but could be carried further to the other components. The communication component receives all changes of the internal states (at best with relevance) and handles the fast and short dialog flow by verbalizing the most critical changes. Currently, we are implementing this architectural model in a first generation of collaborative agents in the CoCu environment.

## 6 Conclusion

In this paper, we introduced and explored the concept of fluid collaboration and its significance in human-human and human-agent interactions. FC is characterized by dynamic and flexible coordination among agents, where tasks and resources are frequently reassigned in response to changing environmental conditions and task demands. Unlike rigid collaborative structures, FC requires agents to adapt quickly, often without explicit communication, relying instead on implicit cues and shared understanding. We provided a compositional definition and characterization of FC, introducing the notions of collaboration units, assignments, and patterns. This framework allows for the characterization and measurement of FC through features such as fluidity of unit assignment, pattern dynamics, and intertwinement. By defining respective metrics, we established quantitative means to assess the dynamics of fluid collaboration.

To study FC between humans and artificial agents empirically, we introduced Cooperative Cuisine, a simulated kitchen environment designed for real-time, fast-paced collaboration between multiple agents. The environment allows for continuous movement and action, enabling the study of both verbal and non-verbal coordination mechanisms. Our exploratory study with human dyads demonstrated that participants naturally engage in FC, dynamically adjusting their assignments and collaboration patterns. These adjustments were often made with minimal explicit communication, highlighting the role of implicit coordination and rapid mentalizing. Our analyses also revealed that participants frequently shifted between different collaboration patterns, adapting to task needs and environmental constraints. We argue that this required continuous monitoring of each other’s actions, intentions, and beliefs, emphasizing the importance of dynamic mentalizing or Theory of Mind (ToM) capabilities for FC. Participants demonstrated the ability to infer partners’ intentions based on minimal cues, integrating perception, planning, and action in a seamless manner. Building on these insights, we discussed foundational steps toward developing FC-capable artificial agents. We emphasized the need for resource-rational mentalizing approaches that balance computational efficiency with inference accuracy. By proposing an architectural framework that integrates satisficing mentalizing and action-driven ToM with hierarchical planning models, we aim to enable agents to engage in FC by dynamically adjusting their mentalizing processes based on situational demands.

Our work contributes to a deeper understanding of the cognitive mechanisms underlying FC and paves the way for designing collaborative agents capable of participating in fluid, dynamic interactions with humans. Future work includes further development of the proposed agent architecture and the full implementation of FC-capable agents in the CoCu environment. Additional studies are needed to examine the impact of different factors on fluidity, such as varying levels of uncertainty, asymmetries in perception and capabilities among agents, and the role of communication modalities. A precise measure of fluidity of a collaboration will be developed based on the established concept of patterns, but must also consider the quality of collaboration patterns that are created and adjusted to demarcate it, e.g., from chaotic interactions with fully random transitions. Understanding how to trigger, control, and enhance fluidity in collaboration will represent a significant step toward more natural and intuitive interactions between humans and artificial agents. By harnessing dynamic mentalizing and adaptive coordination mechanisms, agents can thus become more effective partners in complex, real-world tasks.

## Data Availability

The raw data supporting the conclusions of this article will be made available by the authors, without undue reservation.

## Footnotes

1
https://cdn.worktechacademy.com/uploads/2018/10/WORKTECH-Academy-Avocor-Fluid-Collaboration.pdf

2
https://medium.com/@lovethjen/4b7521b4899a

3 See https://www.team17.com/games/overcooked/, https://www.team17.com/games/overcooked-2/

4 If this were not the case, (nearly) no collaboration would also achieve a seemingly good score.

5 Source code available at https://scs.techfak.uni-bielefeld.de/cooperative-cuisine
